# Violacein induces cell death by triggering mitochondrial membrane hyperpolarization *in vitro*

**DOI:** 10.1186/s12866-015-0452-2

**Published:** 2015-06-06

**Authors:** Angélica Maria de Sousa Leal, Jana Dara Freires de Queiroz, Silvia Regina Batistuzzo de Medeiros, Tatjana Keesen de Souza Lima, Lucymara Fassarella Agnez-Lima

**Affiliations:** Departamento de Biologia Celular e Genética, Universidade Federal do Rio Grande do Norte, CEP 59072-970, Natal, RN Brazil; Departamento de Biologia Celular e Molecular, Centro de Biotecnologia, Universidade Federal da Paraíba, CEP 58051-900, João Pessoa, PB Brazil

**Keywords:** Violacein, *Chromobacterium violaceum*, Oxidative stress, Cytotoxicity, Mitochondrial membrane potential

## Abstract

**Background:**

Violacein is a purple pigment from *Chromobacterium violaceum* that possesses diverse biological and pharmacological properties. Among these, pro-oxidant and antioxidant activities have been suggested. However, the cytotoxic mechanisms induced by violacein are poorly understood and the improvement in knowledge regarding these cell death mechanisms will be useful to develop new therapeutic approaches. Considering this, in our work, we investigated the pro-oxidant effects of violacein in non-tumor (CHO-K1 and MRC-5) and tumor (HeLa) cell lines, searching for a better understanding of reactive oxygen species (ROS) production and cell death induction.

**Results:**

Cytotoxicity induced by violacein was observed in the three cell lines; however, MRC-5 and HeLa cells were shown to be more sensitive to violacein treatment. Although punctual alterations in the antioxidant apparatus and increase in oxidative stress biomarkers was observed in some violacein concentrations, no association was found between increased oxidative stress and induction of cell death. However, the increase of mitochondrial membrane potential was observed.

**Conclusions:**

In fact, the increase of mitochondrial membrane potential in MRC-5 and HeLa cells suggests that mitochondrial membrane hyperpolarization might be the main cause of cell death triggered by violacein.

## Background

Violacein is a purple pigment produced by *Chromobacterium violaceum*, a betaproteobacteria commonly found in tropical and sub-tropical regions. This pigment has attracted interest owing to its several biological and pharmacological activities, including antibiotic [1–3], antitumoral [4–6], antileishmanial [7], antiviral [8, 9], antiprotozoal and antiparasitary [10–12], immunomodulatory, analgesic and antipyretic [13], anti-diarrheal and ulcer-protective effects [14].

The antioxidant activity of violacein has been studied in distinct models, as a scavenger of nitrogen reactive species, 2,2-diphenyl-1-picrylhydrazyl (DPPH) radicals and hydroxyl radicals and by inhibiting lipid peroxidation [15]. Despite this *in vitro* antioxidant potential, ROS production mediated by violacein, followed by activation of caspase-3, release of cytochrome c, calcium release to the cytosol and apoptotic cell death, were reported in colon cancer Caco-2 cells [16]. Moreover, the cytotoxicity toward EAT cells mediated by ROS production and the decrease in intracellular GSH levels were observed after treatment with violacein [5].

Concerning these two contrasting effects (antioxidant and pro-oxidant) and the limited number of cell lines evaluated to date, the present study was carried out to investigate the pro-oxidant effects of violacein in non-tumor and tumor cell lines, aiming to perform a comparative analysis of the cellular responses and a better understanding of the mechanisms involved with cell death that may be useful for developing new therapeutic products.

## Results

### Violacein induced loss of cell viability and cell death by necrosis or apoptosis

**Fig. 1:**
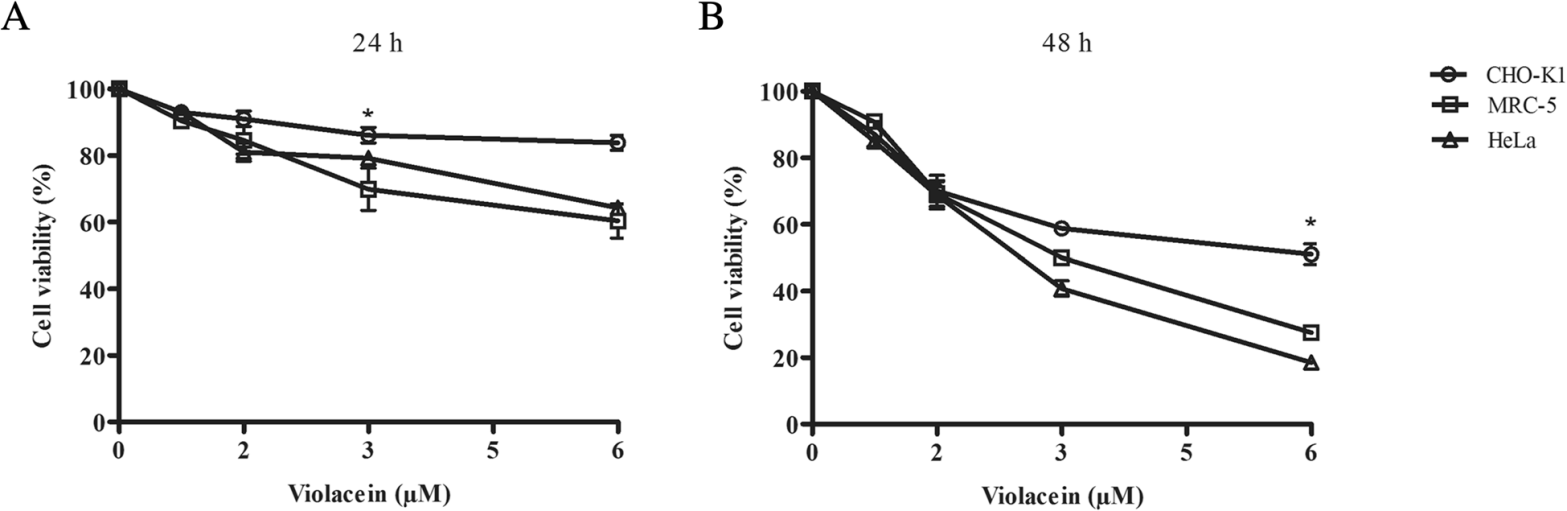
Effects of violacein on the cell viability of CHO-K1, MRC-5 and HeLa cells, as determined by the Trypan blue dye exclusion method after exposure to 0.75–6 μM violacein for 24 (**a**) and (**b**) 48 h. The viability of untreated cells was expressed as 100 %

Thecell viability data obtained using the Trypan blue dye exclusion method showed that, after incubation with violacein for 24 h, MRC-5 and HeLa cells exhibited nearly 60 % of cell viability when exposed to 6 μM violacein. However, a weaker cytotoxicity was observed in CHO-K1 cells (Fig. 1a). As shown in Fig. 1b, exposure to 3 μM violacein for 48 h caused an approximately 50 % decrease in cell viability in all of the cell lines tested, with MRC-5 and HeLa cells being more sensitive to the treatment.

**Fig. 2:**
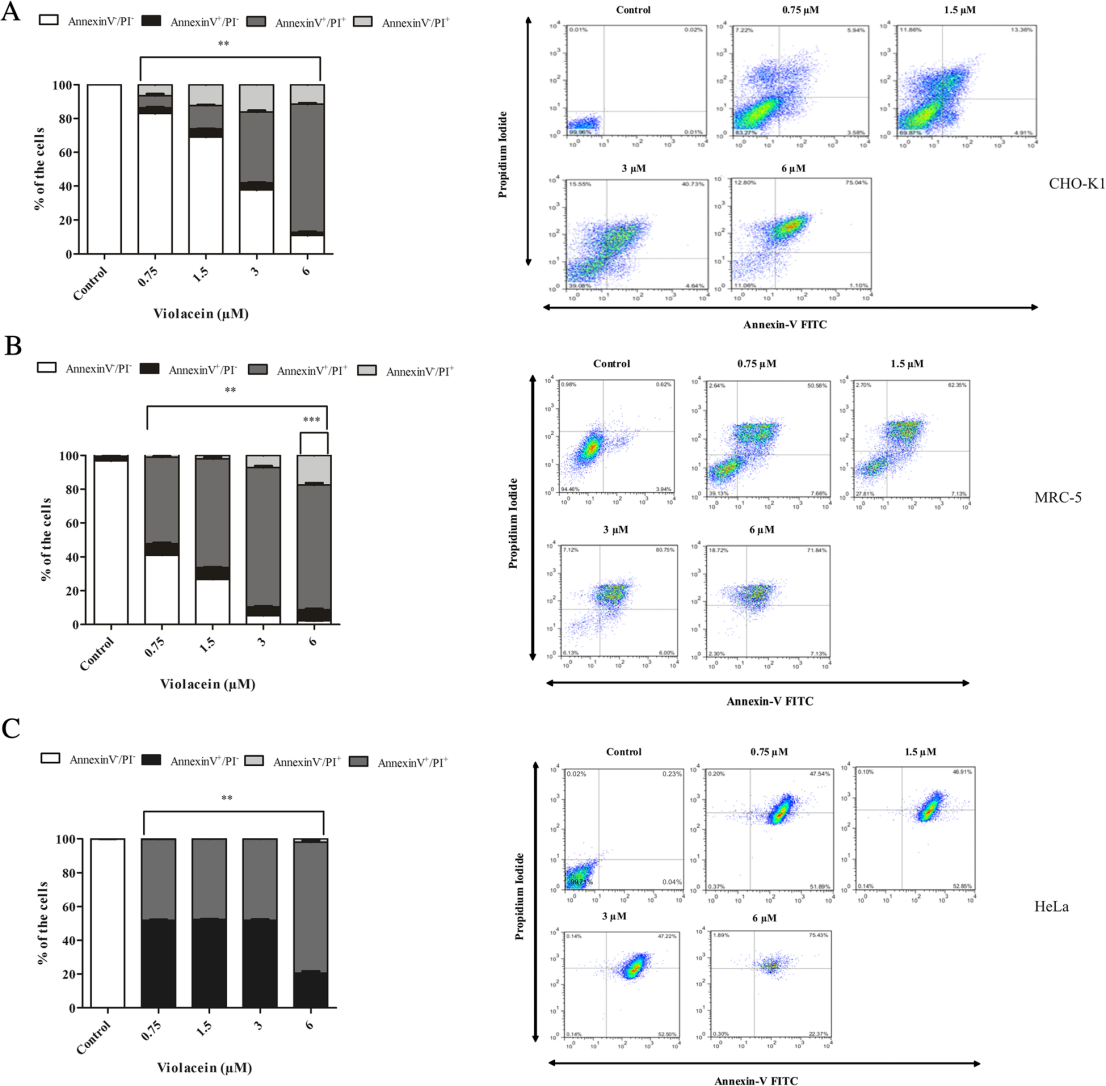
Cells death induced by violacein determined by flow cytometry. The percentage of CHO-K1 (**a**), MRC-5 (**b**) and HeLa cells (**c**) exposed to 0.75–6 μM violacein presenting externalization of phosphatidylserine in the outer layer are shown. The Annexin V^+^/P^−^ cells plus Annexin V^+^/PI^+^ cells were detected as apoptotic cells. Annexin V^−^/PI^+^ cells were labeled as necrotic cells. All of the results are expressed as the means ± SD of three independent experiments (*p < 0.05 CHO-K1 vs. MRC-5 and HeLa cells; **p < 0.001 treated vs. control; ***p < 0.001 AnnexinV^−^/PI^+^ versus AnnexinV^−^/PI^−^ cells exposed to 6 μM violacein)

Annexin V is a recombinant phosphatidylserine-binding protein that specifically interacts with phosphatidylserine residues and can be used for the detection of apoptosis. Cells treated with violacein were stained with Annexin-V and PI for necrosis detection [17, 18]. In all of the concentrations tested, violacein could induce necrosis in CHO-K1 cells due to the significant increase (*p* < 0.001) in AnnexinV^−^/PI^+^ and AnnexinV^+^/PI^+^ cells (Fig. 2a). Although AnnexinV^+^/PI^+^ is characteristic of both late apoptotic or early necrotic cells, the low percentage of AnnexinV^+^/PI^−^ labeling, which is characteristic of early apoptotic cells, might be an indication that cells presenting double staining (AnnexinV^+^/PI^+^) are undergoing necrosis. For MRC-5 cells, labeling features of both necrotic and apoptotic cells were observed with all of the concentrations tested (Fig. 2b). However, the percentage of necrotic cells was significant with 6 μM violacein, suggesting that necrosis might be the predominant cell death mechanism. By contrast, the results obtained with HeLa cells showed that most of the cells displayed markers of early and late apoptosis when exposed to violacein (Fig. 2c). As observed in the Trypan blue dye exclusion assays (Fig. 1) and in the flow cytometric analysis, the decrease in cell viability was not continuously observed, as higher concentrations were tested. However, there were differences between given concentrations. Fig. 2a-c also shows the dot plots of Annevin V/FITC against PI (panels at right).

### Effects on the antioxidant apparatus

**Fig. 3:**
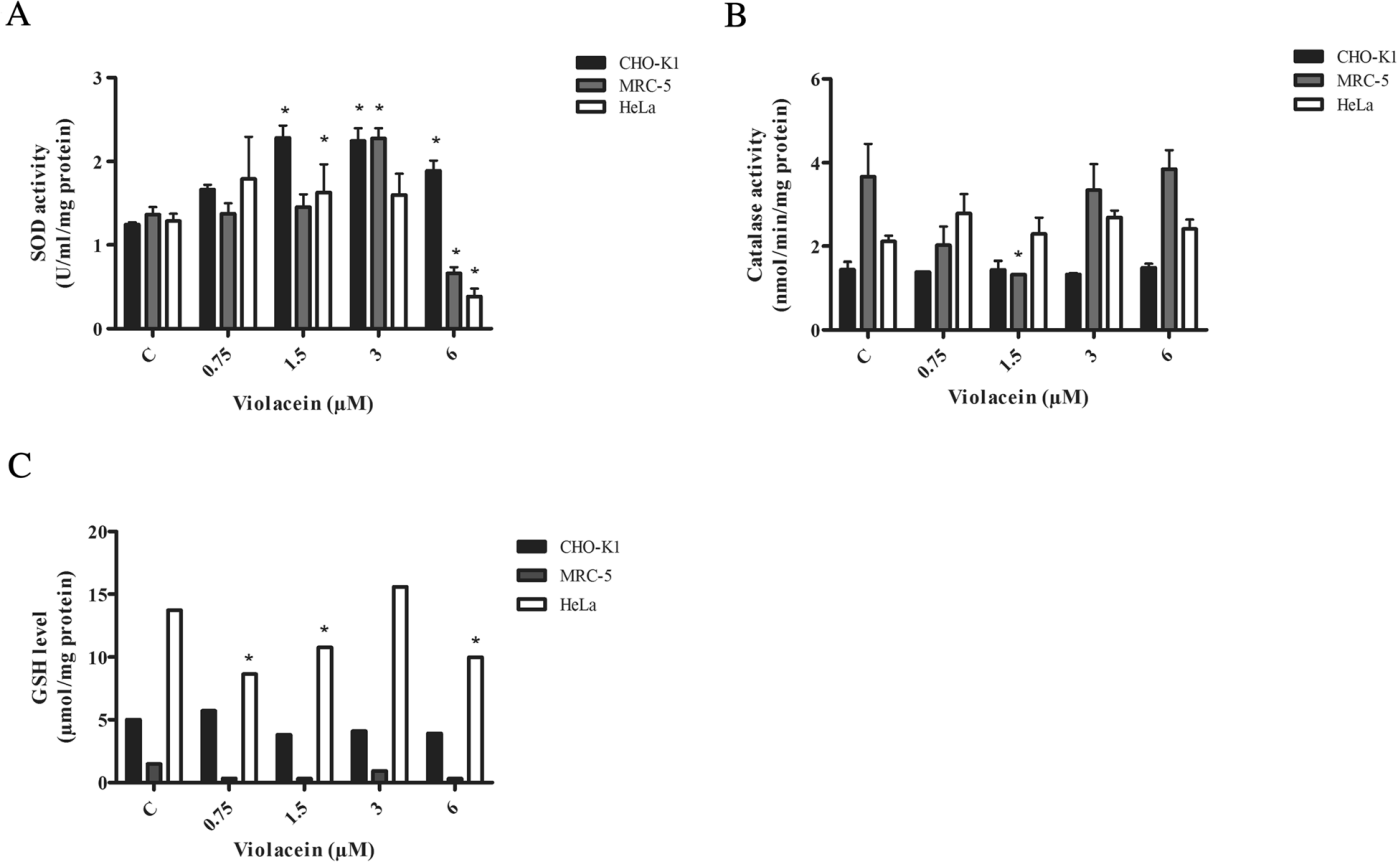
Status of the antioxidant apparatus after violacein treatment. **a** SOD activity in CHO-K1, MRC-5 and HeLa cells exposed to 0.75–6 μM violacein for 24 h. SOD activity was expressed as U/mL/mg protein. Standard controls using SOD yielded 15.4 U/mL/mg protein of enzymatic activity. **b** Catalase activity in CHO-K1, MRC-5 and HeLa cells exposed to 0.75–6 μM violacein for 24 h. Catalase activity was expressed as nmol/min/mg protein. Standard controls using CAT yielded 12.3 nmol/min/mg protein of enzymatic activity. **c** Alterations in the GSH levels of CHO-K1, MRC-5 and HeLa cells exposed to 0.75–6 μM violacein for 24 h. The results are presented as the means ± SD of three experiments run in triplicate (*p < 0.05 treated vs. control). C denotes control

As demonstrated in Fig.3a, in CHO-K1 cells, violacein at 1.5-6 μM led to an increase in SOD activity. Regarding to MRC-5 cells, a significant increase in SOD levels was observed specifically in cells exposed to violacein at 3 μM, but a decreased SOD activity was observed when cells were submitted to 6 μM. In fact, the decrease was observed either for HeLa cells submitted to violacein at 6 μM and one possible explanation for this decrease might be violacein cytotoxicity at this concentration. Considering the catalase assays, not even the highest concentration tested caused significant changes in enzymatic activity in CHO-K1 and HeLa cells. In contrast, in MRC-5 cells, violacein at 1.5 μM lead to the inhibition of catalase activity, but it is not clear why this occurs (Fig. 3b). A significant decrease in GSH levels was observed in HeLa cells exposed to violacein at 0.75, 1.5 and 6 μM (Fig. 3c).

### Oxidative stress biomarker detection

**Fig. 4:**
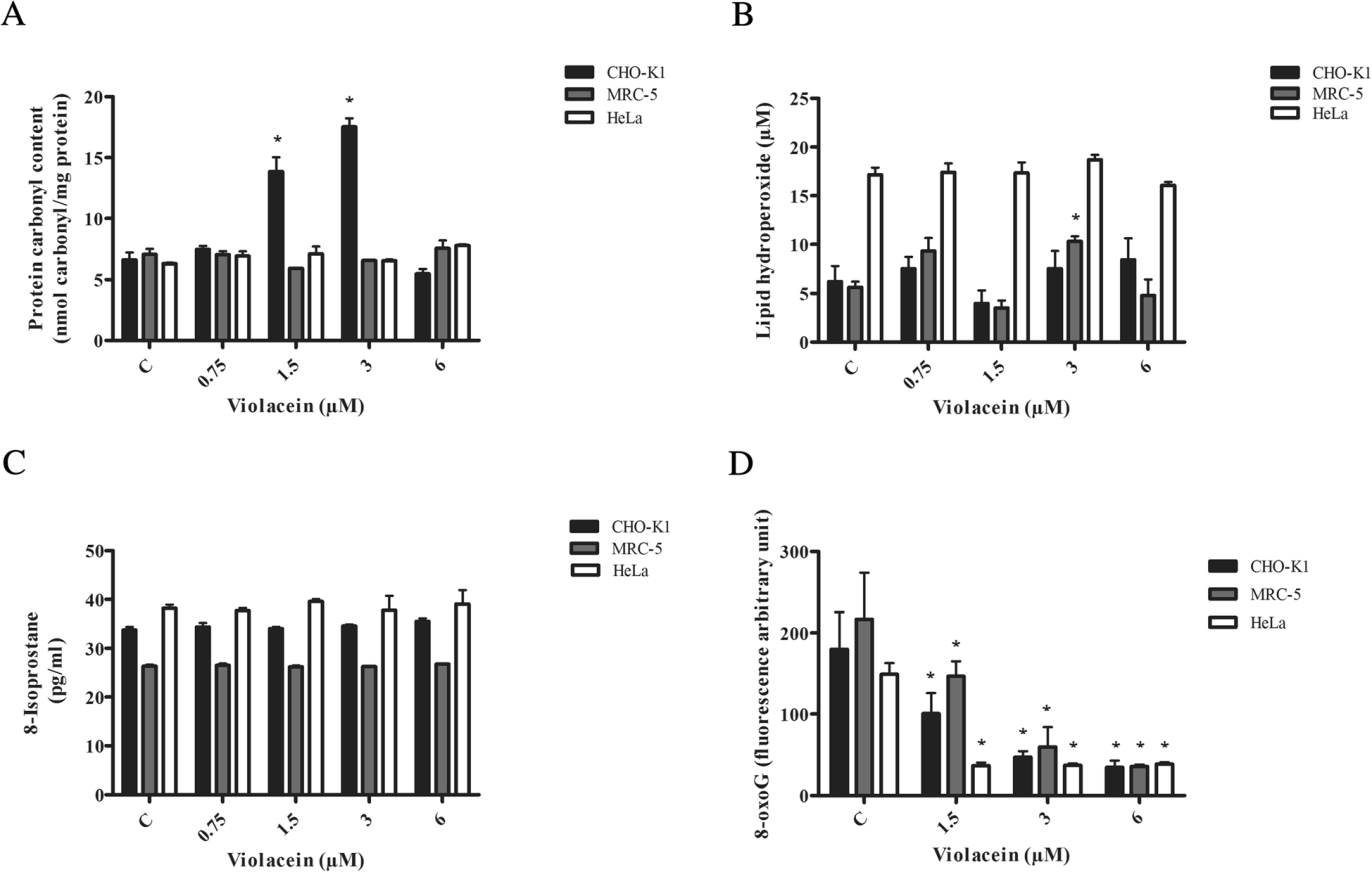
Analysis of biomarkers of oxidative stress after treatments with violacein. **a** Protein carbonylation in CHO-K1, MRC-5 and HeLa cells exposed to 0.75–6 μM violacein for 24 h. **b** Lipid hydroperoxide (LPO) levels (μM) in CHO-K1, MRC-5 and HeLa cells exposed to 0.75–6 μM violacein 24 h. **c** 8-iso PGF2a levels (pg/mL) in CHO-K1, MRC-5 and HeLa cells exposed to 0.75–6 μM violacein for 24 h. **d** 8-oxoG quantification in CHO-K1, MRC-5 and HeLa cells exposed to 0.75–6 μM violacein for 24 h. The results are expressed as the means ± SD of three experiments run in triplicate (*p < 0.05 treated vs. control). C denotes control

A significant increase in the protein carbonyl levels was observed exclusively in CHO-K1 cells exposed to 1.5–3 μM violacein (Fig. 4a). Regarding to lipid peroxidation markers, increased lipid hydroperoxide levels were observed in MRC-5 cells exposed to 3 μM violacein (Fig.4b). In contrast, violacein was not able to increase the levels of 8-isoprostanes in any concentration tested (Fig. 4c). Interestingly, a decrease in 8-oxoguanine (8-oxoG, a DNA oxidized lesion) levels was observed in CHO-K1, MRC-5 and Hella cells exposed to 1.5-6 μM violacein (Fig. 4d).

### Effect of violacein on mitochondrial membrane potential

**Fig. 5:**
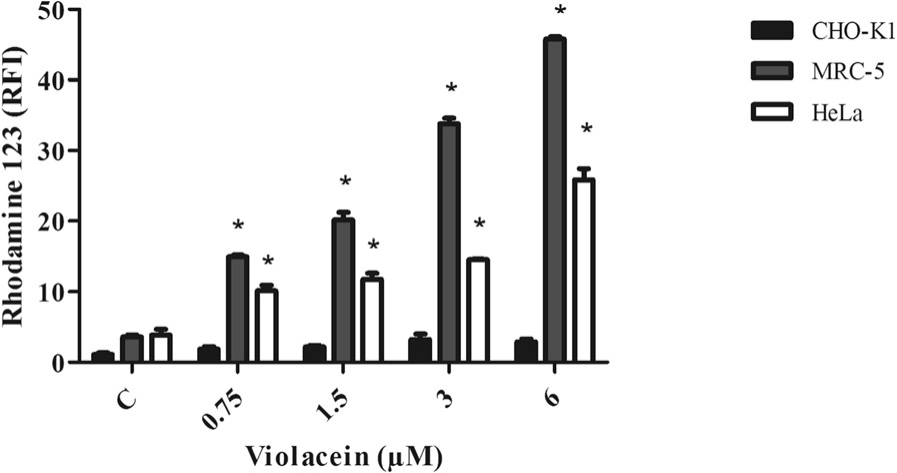
Effects of violacein on the mitochondrial membrane potential of CHO-K1, MRC-5 and HeLa cells. Cells were stained with Rhodamine 123 and analyzed by flow cytometry in the FL-1 (FITC) channel. The results are expressed as the means ± SD of three experiments run in triplicate (*p < 0.05 treated vs. control). C denotes control

Considering that RH123 uptake is proportional to the mitochondrial transmembrane potential, the results shows that violacein leads to the increase of the mitochondrial membrane potential (Fig. 5) in MRC-5 and HeLa cells at all of the concentrations tested. By contrast, in CHO-K1 cells, no significant changes in the membrane potential were detected. These data suggest that the cytotoxicity induced by violacein may be due to mitochondrial damage that leads to membrane hyperpolarization, triggering cell death. However, the cytotoxicity of violacein in CHO-K1 cells may be related to a violacein cell-type specific mechanisms, other than the induction of mitochondrial membrane hyperpolarization.

## Discussion

In general, the cytotoxicity of violacein for non-tumor cells occurs in the range of 5–10 μM [4, 5, 19], while the cytotoxicity for tumor cells is observed in the range of 1–5 μM [5, 16, 20], findings that are in agreement with the data obtained in our work. Concerning tumor cells, the most promising results were obtained with the cell lines MOLT-4 (leukemia), NCI-H460 (lung cancer) and KM12 (colon cancer), in which very low violacein concentrations (0.03–0.06 μM) showed cytotoxicity [4]. Moreover, in melanoma 92.1andOCM-1 cells, the cytotoxicity was exhibited in the range of 1.69–3.69 μM [20]. In our work, violacein cytotoxicity was highest at 6 μM. Indeed, variations in violacein cytotoxicity are observed depending on the cell type, indicating the occurrence of violacein cell-type-specific mechanisms [5, 16].

Previously, the cytotoxicity induced by violacein was attributed to ROS generation [5, 16], despite its antioxidant activity observed *in vitro* [15]. Here, we analyzed the induction of antioxidant enzymes and occurrence of oxidative stress biomarkers in cells treated with violacein to identify the association between oxidative stress and cell death. We observed that certain concentrations of violacein induced SOD activity in CHO-K1 and MRC-5 cells, but a dose-dependent response was not obtained. Concerning catalase activity, significant differences were not observed. Interestingly, catalase activity was reduced in MRC-5 cells after the treatment with 1.5 μM violacein, but the cause of this inhibition is unclear. Despite the relationship between SOD and catalase activities [21], the non-concomitant increase in the activity of these enzymes has been demonstrated [22], as observed in our work.

According to Bromberg *et al*. [5], treatment with violacein (2–5 μM) led to the reduction of GSH levels in EAT cells. Similarly, in our work, a significant decrease in GSH levels was observed in HeLa cells treated with specific concentrations of violacein. However, the GSH depletion in HeLa cells may be caused by mechanisms other than an excessive level of ROS. In fact, we observed the induction of SOD and occurrence of oxidative stress biomarkers for certain concentrations of violacein (Figs. 3 and 4). However, these data do not support the role of ROS in the cell death observed after treatment because dose-dependent responses were not observed.

Interestingly, the increase of membrane potential was detected even at the lowest violacein concentration tested, suggesting that violacein toxicity is associated with mitochondria damage. Comparing these results with the cytotoxicity induced by violacein, the co-occurrence of cytotoxicity and the increase of mitochondrial membrane potential might be indicative that cytotoxicity caused by violacein occurs through the mitochondrial pathway. However, the differences observed in cell viability and the proportions of cells in apoptosis and/or necrosis (Fig. 2) suggest the occurrence of cell-type-specific mechanisms. Previous study [23] carried out with 2,237 fibrosarcoma cells treated for 48 h with PVP, a violacein-like pigment from the Antarctic *Janthinobacterium sp* bacteria, showed that the treatment induced the disruption of the mitochondrial membrane potential and occurrence of apoptosis via the mitochondrial pathway. Furthermore, another cell death mechanism has been suggested by Queiroz *et al*. [6], showing the effect of violacein on TF1 leukemia cells by the induction of cellular suicide, by a mechanism involving the endoplasmic reticulum, Golgi linearization and ‘horseshoe-shaped’ nuclei. Melo *et al.* demonstrated that the treatment of HL60 cells with violacein induced cytotoxic effects and cell differentiation, which may be related to alterations in phospholipid asymmetry and changes in mitochondrial polarization [24]. In addition, the violacein-dependent association of TRAF2 with the TNF receptor was observed by co-immunoprecipitation assays, suggesting that apoptosis of HL60 cells mediated by violacein occurs by specific activation of TNF receptor 1 [25].

Mitochondrial dysfunction has been shown to participate in the induction of cell death and has been suggested to be central to the apoptotic pathway [26]. Indeed, early hyperpolarization of mitochondrial membrane has been reported as an event that occurs in several cell death pathways [27–29]. Collectively, the results suggest that violacein induces cell death of both MRC-5 and HeLa cells through the hyperpolarization of the mitochondrial membrane potential, acting through a mitochondrial pathway. By contrast, in CHO-K1 cells, the cytotoxic effects of violacein may be related to other mechanisms specific for this cell line.

## Conclusion

In conclusion, although violacein induced SOD activity and increased certain oxidative stress biomarkers, oxidative stress does not seem to be the major cause of cell death observed after the treatment because dose-dependent responses were not observed. Interestingly, in MRC-5 and HeLa cells, the hyperpolarization of the mitochondrial membrane potential may be related to cell death triggered by violacein. Due to the pharmacological potential of violacein, a better understanding of the cell death mechanisms induced by this compound will be useful to develop new therapeutic approaches.

## Methods

### Violacein

Purified violacein (3-(1,2-dihydro-5-(5-hydroxy-1H-indol-3-yl)-2-oxo-3H-pyrrol-3-ilydene)-1,3-dihydro-2H-indol-2-one) [9, 10, 30] was kindly provided by Dr. Regina Vasconcellos Antonio (BQM-UFSC, Brazil). Violacein was dissolved in DMSO (dimethyl sulfoxide; Merck, Germany) and added to the cultured medium to a final DMSO concentration of less than 0.1 % (vol/vol). The violacein was stored at 4 °C, protected from light and diluted in fresh cell culture medium prior to use.

### Cytotoxicity assays

To evaluate the cytotoxic effects of violacein, MRC-5 (human fetal lung fibroblast), CHO-K1 (Chinese hamster ovary) and HeLa (human cervical adenocarcinoma) cells were cultured in flasks containing DMEM medium (Gibco, USA) supplemented with 10 % fetal bovine serum (FBS) and antibiotics (100 U/mL of penicillin and 100 μg/mL of streptomycin) (Gibco) in a CO_2_ incubator at 37 °C. For the cytotoxicity assays, cells were seeded (3 × 10^5^ cells mL^−1^/well) in 6-well plates for 24 h and exposed to 0.75–6 μM violacein for 24 and 48 h. Next, the cell monolayers were washed three times with cold PBS, pH 7.4, harvested using a 1× trypsin-EDTA solution (Gibco) and resuspended in fresh DMEM medium. Cell viability was measured using the Trypan blue dye exclusion method [31].

To evaluate the occurrence of cell death by apoptosis or necrosis, the cells were treated as described above. After the treatments, cells were briefly washed in cold PBS, pH 7.4, and collected by trypsinization. The cell pellets were resuspended in a binding buffer (1 × 10^6^/mL cell density) and stained with 5 μL of Annexin V-FITC and 5 μL of propidium iodide (PI), as indicated in the FITC Annexin V Apoptosis Detection Kit I (Catalog No.556547; BD Pharmingen^TM^, USA). The cells were incubated in the dark at room temperature for 15 min and analyzed on a FACSCANTO II flow cytometry system (BD Biosciences, USA). For each sample, 30,000 events were collected, and the analysis was performed using Flowjo 7.6.4 software (Treestar Ashland, USA).

### SOD and Catalase activities

Violacein-exposed cells were washed twice by resuspension in cold PBS, pH 7.4, and the cell pellets were sonicated in a cold buffer (20 mM HEPES buffer (Amresco, USA), pH 7.2, containing 1 mM EGTA (ethylene glycol tetraacetic acid; Sigma-Aldrich, USA), 210 mM mannitol and 70 mM sucrose) using the Ultrasonic processor (Sonic vibraCell^TM^, Canada). SOD activity was evaluated as indicated in the Superoxide Dismutase Assay Kit (Catalog No.706002; Cayman Chemical Company, USA). For catalase activity measurements, cells were treated, washed twice in PBS, pH 7.4, sonicated in a cold buffer (50 mM potassium phosphate, pH 7.0, containing 1 mM EDTA) and tested for enzymatic activity as indicated in the Catalase Assay Kit (Catalog No.707002; Cayman Chemical Company). Protein quantification was determined by the Bradford assay [32].

### GSH levels

To determine whether violacein can reduce cellular GSH levels, cells were washed twice in cold PBS, pH 7.4, and the cell pellets were homogenized in a cold buffer (50 mM MES, pH 7.0, containing 1 mM EDTA). The supernatant was stored on ice and deproteinized using metaphosphoric acid and triethanolamine at 0.1 g/mL and 4 M, respectively. Deproteinized samples were used to assay total GSH (both oxidized and reduced forms) as indicated in the Glutathione Assay Kit (Catalog No. 703002, Cayman Chemical Company). The normalization was based the protein content of samples as determined by the Bradford assay [32].

### Membrane lipid peroxidation assays: Lipid hydroperoxides (LPOs) and 8-Isoprostanes (8-iso PGF2a)

After incubation with violacein, cells were washed twice by resuspension in cold PBS, pH 7.4, and the cell pellets were sonicated in HPLC-grade water. The LPOs and (8-iso PGF2a) were extracted and quantified as indicated in the Lipid Hydroperoxide Assay Kit (Catalog No. 705003; Cayman Chemical Company) and 8-Isoprostane EIA Kit (Catalog No.516351, Cayman Chemical Company), respectively.

### Protein carbonyls

Total protein was extracted as described in [33] with modifications. Cells were centrifuged, collected in cold PBS and incubated in lysis buffer (50mMTris-HCl, 150 mM NaCl, 0.1 % SDS, HPLC-ultrapure grade water, protease inhibitors (1:100) (Merck) and 25 U DNase benzonase (Merck) for 20 min at 4 °C. After lysis, the supernatants containing total protein extracts were recovered and the proteins quantified by the Bradford method [32]. After that, protein carbonyls were quantified as proposed by Hawkins *et al*. [34].

### Detection of 8-oxoguanine

Initially, cells at 10^5^ cells mL^−1^/well density were seeded in 96-well plates for 24 h and treated with violacein. Next, cells were fixed in methanol at −20 °C (Labsynth) for 20 min, washed in cold PBS and permeabilized with PBS containing 0.1 % Triton X-100 (USB corporation). Nonspecific ligation events were blocked by incubating cells in PBS containing 10 % FBS for 1 h. Finally, the cells were incubated in the dark at 37 °C in PBS containing FITC-avidin (Sigma-Aldrich) (1:200 dilution) for 1 h. After four washes in PBS containing 10 % FBS, fluorescence was detected at 485 nm excitation and 535 emission (GloMax®-Multi Detection System; Promega, USA).

### Mitochondrial dysfunction evaluation

Changes in the mitochondrial transmembrane potential were assayed through the incorporation of Rhodamine 123 (Rh123) (Sigma-Aldrich), a mitochondrial-specific cationic fluorescent dye. After treatments, cells were resuspended at 1 × 10^5^/mL in cold PBS and permeabilized with methanol at −20 °C (Labsynth) and incubated with 10 μg/mL of Rh123 (Sigma-Aldrich) for 30 min at 37 °C in the dark. For fluorescence-activated cell sorter (FACS) analysis, 30,000 events for each sample were acquired in the FACSCANTO II system (BD Biosciences), at wavelengths of 488 nm for excitation and 525 nm for emission as proposed by Li *et al*. [35]. The analysis was performed using Flowjo 7.6.4 software (Treestar Ashland, USA).

### Statistical analysis

Values are presented as the means ± standard deviation (SD) of three independent experiments, and statistical significance was set as a *p* value less than 0.05. Significance was determined by analysis of variance followed by the Dunnett’s or Bonferroni post-hoc test (GraphPad Prism 5.0 program).

## Abbreviations

SOD: Superoxide dismutase
DPPH: 2,2-diphenyl-1-picrylhydrazyl
ROS: Reactive oxygen species
EAT: Ehrlich ascites tumor
GSH: Glutathione
DMSO: Dimethyl sulfoxide
DMEM: Dulbecco’s modified Eagle’s medium
FBS: Fetal bovine serum
PBS: Phosphate balanced salt solution
HEPES: 4-(2-hydroxyethyl)-1-piperazineethanesulfonic acid
EGTA: Ethylene glycol tetraacetic acid
EDTA: Ethylene diamine tetraacetic acid
CAT: Catalase
LPO: Lipid hydroperoxide
EIA: Enzyme immunoassay
8-Isoprostanes: (8-iso PGF2a)
SDS: Sodium dodecyl sulfate
DNPH: 2,4-Dinitrophenylhydrazine
PGF2a: Prostaglandin F2 alpha
EC-SOD: Extracellular superoxide dismutase
GPx: Gluthatione peroxidase
